# 3-Chloro-6-(3,5-dimethyl-1*H*-pyrazol-1-yl)picolinic acid–triphenyl­phosphine oxide (1/1)

**DOI:** 10.1107/S160053680802045X

**Published:** 2008-07-09

**Authors:** Fei-Long Hu, Zhong-Jing Huang, Shan-Shan Zhang, Yue Zhuang, Wei-Qiang Luo

**Affiliations:** a College of Chemistry and Ecological Engineering, Guangxi University for Nationalities, Nanning 530006, People’s Republic of China

## Abstract

In the title 1:1 adduct, C_11_H_10_ClN_3_O_2_·C_18_H_15_OP, the dihedral angle between the pyridine and pyrazole rings is 10.3 (2)°. The two components of the adduct are linked by an O—H⋯O hydrogen bond.

## Related literature

For background, see: Mann *et al.* (1992 ▶).
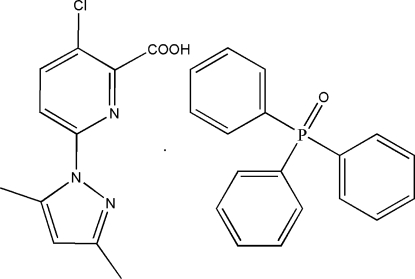


## Experimental

### 

#### Crystal data

C_11_H_10_ClN_3_O_2_·C_18_H_15_OP
*M* *_r_* = 529.94
Monoclinic, 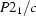

*a* = 16.6694 (14) Å
*b* = 9.8176 (11) Å
*c* = 18.272 (2) Å
β = 116.089 (2)°
*V* = 2685.7 (5) Å^3^
*Z* = 4
Mo *K*α radiation
μ = 0.24 mm^−1^
*T* = 298 (2) K
0.23 × 0.18 × 0.09 mm

#### Data collection

Bruker SMART CCD diffractometer
Absorption correction: multi-scan (*SADABS*; Sheldrick, 1996 ▶) *T*_min_ = 0.947, *T*_max_ = 0.979
13279 measured reflections
4721 independent reflections
2333 reflections with *I* > 2σ(*I*)
*R*_int_ = 0.063

#### Refinement

*R*[*F* ^2^ > 2σ(*F* ^2^)] = 0.045
*wR*(*F* ^2^) = 0.083
*S* = 1.03
4721 reflections
337 parameters
H atoms treated by a mixture of independent and constrained refinement
Δρ_max_ = 0.18 e Å^−3^
Δρ_min_ = −0.27 e Å^−3^

### 

Data collection: *SMART* (Siemens, 1996 ▶); cell refinement: *SAINT* (Siemens, 1996 ▶); data reduction: *SAINT*; program(s) used to solve structure: *SHELXS97* (Sheldrick, 2008 ▶); program(s) used to refine structure: *SHELXL97* (Sheldrick, 2008 ▶); molecular graphics: *SHELXTL* (Sheldrick, 2008 ▶); software used to prepare material for publication: *SHELXTL*.

## Supplementary Material

Crystal structure: contains datablocks I, global. DOI: 10.1107/S160053680802045X/hb2757sup1.cif

Structure factors: contains datablocks I. DOI: 10.1107/S160053680802045X/hb2757Isup2.hkl

Additional supplementary materials:  crystallographic information; 3D view; checkCIF report

## Figures and Tables

**Table 1:** Hydrogen-bond geometry (Å, °)

*D*—H⋯*A*	*D*—H	H⋯*A*	*D*⋯*A*	*D*—H⋯*A*
O1—H1⋯O3	0.79	1.76	2.537 (2)	165

**Fig. 1.**
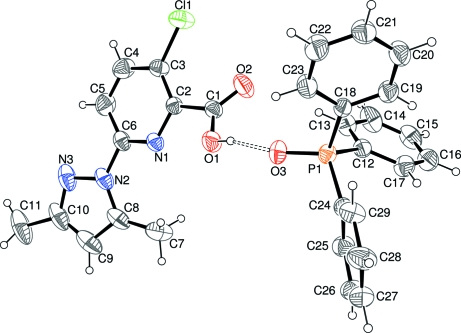
The molecular structure of (I) showing 30% probability displacement ellipsoids for the non-hydrogen atoms. The hydrogen bond is indicated by a double-dashed line.

## supplementary crystallographic information

### Comment

Pyrazoles have been investigated extensively, owing to their chelating ability with metal ions and their potentially beneficial biological activities (e.g. Mann *et al.*, 1992). As part of our studies on these compounds, we report here the synthesis and crystal structure of the title compound, (I), (Fig. 1).

In the 3-chloro-6-(3,5-dimethyl-1*H*-pyrazol-1-yl)picolinic acid molecule, the dihedral angle between the two ring mean planes is 10.3 (2) °.  The two components of the adduct interact by way of an O—H···O hydrogen bond (Table 1).

### Experimental

3-Chloro-6-(3,5-dimethyl-1*H*-pyrazol-1-yl)picolinic acid (1 mmol, 251.04 mg) was dissolved in distilled water (15 ml) and triphenylphosphine oxide (0.5 mmol, 139.04 mg) in distilled water (5 ml) was added with stirring at 323 K. The resulting solution was allowed to react for 5 h and was then filtered. Colourless blocks of (I) were obtained by slow evaporation of a water solution over a period of one month (yield 75%). Elemental analysis: found: C 65.71; H 4.73; N 7.94; O 9.06%. calc. for C_29_H_25_ClN_3_O_3_P: C 65.72; H 4.75; N 7.93; O 9.06%.

### Refinement

The C-bound H atoms were positoned geometrically (C—H = 0.93-0.96Å) and refined as riding with *U*_iso_(H) = 1.2*U*_eq_(C) or 1.5*U*_eq_(methyl C). The O-bound H atom was located in a difference map and refined as riding in its as-found relative position with *U*_iso_(H) =1.5*U*_eq_(O).

### Crystal data

**Table:**

C_11_H_10_ClN_3_O_2_·C_18_H_15_OP	*F*_000_ = 1104
*M**_r_* = 529.94	*D*_x_ = 1.311 Mg m^−^^3^
Monoclinic, *P*2_1_/*c*	Mo *K*α radiation λ = 0.71073 Å
Hall symbol: -P 2ybc	Cell parameters from 2022 reflections
*a* = 16.6694 (14) Å	θ = 2.3–25.2º
*b* = 9.8176 (11) Å	µ = 0.24 mm^−^^1^
*c* = 18.272 (2) Å	*T* = 298 (2) K
β = 116.089 (2)º	Block, colourless
*V* = 2685.7 (5) Å^3^	0.23 × 0.18 × 0.09 mm
*Z* = 4	

### Data collection

**Table:**

Bruker SMART CCD diffractometer	4721 independent reflections
Radiation source: fine-focus sealed tube	2333 reflections with *I* > 2σ(*I*)
Monochromator: graphite	*R*_int_ = 0.063
*T* = 298(2) K	θ_max_ = 25.0º
ω scans	θ_min_ = 1.4º
Absorption correction: multi-scan(SADABS; Sheldrick, 1996)	*h* = −19→19
*T*_min_ = 0.947, *T*_max_ = 0.979	*k* = −11→11
13279 measured reflections	*l* = −11→21

### Refinement

**Table:**

Refinement on *F*^2^	Secondary atom site location: difference Fourier map
Least-squares matrix: full	Hydrogen site location: inferred from neighbouring sites
*R*[*F*^2^ > 2σ(*F*^2^)] = 0.045	H atoms treated by a mixture of independent and constrained refinement
*wR*(*F*^2^) = 0.083	*w* = 1/[σ^2^(*F*_o_^2^) + (0.0142*P*)^2^] where *P* = (*F*_o_^2^ + 2*F*_c_^2^)/3
*S* = 1.04	(Δ/σ)_max_ = 0.001
4721 reflections	Δρ_max_ = 0.18 e Å^−^^3^
337 parameters	Δρ_min_ = −0.27 e Å^−^^3^
Primary atom site location: structure-invariant direct methods	Extinction correction: none

### Special details

**Table:**

Geometry. All esds (except the esd in the dihedral angle between two l.s. planes) are estimated using the full covariance matrix. The cell esds are taken into account individually in the estimation of esds in distances, angles and torsion angles; correlations between esds in cell parameters are only used when they are defined by crystal symmetry. An approximate (isotropic) treatment of cell esds is used for estimating esds involving l.s. planes.
Refinement. Refinement of F^2^ against ALL reflections. The weighted R-factor wR and goodness of fit S are based on F^2^, conventional R-factors R are based on F, with F set to zero for negative F^2^. The threshold expression of F^2^ > 2sigma(F^2^) is used only for calculating R-factors(gt) etc. and is not relevant to the choice of reflections for refinement. R-factors based on F^2^ are statistically about twice as large as those based on F, and R- factors based on ALL data will be even larger.

### Fractional atomic coordinates and isotropic or equivalent isotropic displacement parameters (Å^2^)

**Table:**

	*x*	*y*	*z*	*U*_iso_*/*U*_eq_	
P1	0.12197 (5)	0.76008 (8)	0.14024 (4)	0.0528 (2)	
O3	0.16936 (11)	0.88630 (18)	0.18229 (10)	0.0645 (5)	
C12	0.18622 (17)	0.6094 (3)	0.18123 (18)	0.0533 (7)	
C13	0.24643 (19)	0.6082 (3)	0.2624 (2)	0.0723 (9)	
H13	0.2538	0.6855	0.2941	0.087*	
C14	0.2960 (2)	0.4923 (4)	0.2970 (2)	0.0887 (11)	
H14	0.3359	0.4920	0.3520	0.106*	
C15	0.2869 (2)	0.3793 (4)	0.2513 (3)	0.0828 (11)	
H15	0.3216	0.3027	0.2745	0.099*	
C16	0.2266 (2)	0.3784 (3)	0.1711 (2)	0.0783 (10)	
H16	0.2190	0.3003	0.1400	0.094*	
C17	0.17677 (19)	0.4932 (3)	0.13616 (18)	0.0683 (9)	
H17	0.1362	0.4919	0.0813	0.082*	
C18	0.01884 (17)	0.7409 (3)	0.14774 (15)	0.0504 (7)	
C19	−0.0251 (2)	0.6190 (3)	0.13663 (17)	0.0683 (9)	
H19	−0.0013	0.5412	0.1247	0.082*	
C20	−0.1048 (2)	0.6108 (3)	0.14302 (19)	0.0800 (10)	
H20	−0.1339	0.5277	0.1362	0.096*	
C21	−0.1400 (2)	0.7247 (4)	0.15932 (19)	0.0793 (10)	
H21	−0.1939	0.7193	0.1627	0.095*	
C22	−0.0977 (2)	0.8459 (4)	0.1707 (2)	0.0884 (11)	
H22	−0.1224	0.9235	0.1818	0.106*	
C23	−0.0169 (2)	0.8536 (3)	0.16571 (18)	0.0732 (9)	
H23	0.0130	0.9364	0.1747	0.088*	
C24	0.09431 (19)	0.7625 (3)	0.03445 (16)	0.0542 (7)	
C25	0.16220 (19)	0.7500 (3)	0.01058 (19)	0.0682 (9)	
H25	0.2203	0.7338	0.0496	0.082*	
C26	0.1448 (2)	0.7614 (3)	−0.0703 (2)	0.0781 (9)	
H26	0.1909	0.7529	−0.0857	0.094*	
C27	0.0600 (3)	0.7849 (3)	−0.1274 (2)	0.0841 (11)	
H27	0.0482	0.7926	−0.1819	0.101*	
C28	−0.0077 (2)	0.7974 (3)	−0.1057 (2)	0.0905 (12)	
H28	−0.0655	0.8133	−0.1453	0.109*	
C29	0.0090 (2)	0.7866 (3)	−0.02499 (19)	0.0721 (9)	
H29	−0.0378	0.7956	−0.0106	0.086*	
O1	0.29030 (12)	1.0307 (2)	0.28946 (12)	0.0745 (6)	
H1	0.2471	0.9901	0.2595	0.112*	
C1	0.32200 (18)	0.9910 (3)	0.36492 (19)	0.0551 (8)	
O2	0.29977 (14)	0.8882 (2)	0.38566 (13)	0.0925 (7)	
C2	0.38830 (17)	1.0884 (3)	0.42273 (18)	0.0496 (7)	
N1	0.43821 (15)	1.1548 (2)	0.39350 (13)	0.0558 (6)	
C3	0.39748 (18)	1.1108 (3)	0.49982 (19)	0.0620 (8)	
Cl1	0.33173 (5)	1.03306 (10)	0.53878 (5)	0.0911 (3)	
C4	0.4605 (2)	1.2043 (4)	0.5487 (2)	0.0834 (11)	
H4A	0.4674	1.2218	0.6011	0.100*	
C6	0.49929 (19)	1.2413 (3)	0.4415 (2)	0.0610 (8)	
N2	0.55065 (16)	1.3065 (3)	0.4085 (2)	0.0720 (8)	
C5	0.5116 (2)	1.2697 (3)	0.5199 (2)	0.0819 (10)	
H5A	0.5542	1.3326	0.5521	0.098*	
N3	0.60429 (19)	1.4127 (3)	0.45224 (19)	0.0929 (10)	
C8	0.5594 (2)	1.2799 (4)	0.3382 (3)	0.0800 (11)	
C7	0.5137 (2)	1.1695 (4)	0.27913 (19)	0.0996 (12)	
H7A	0.5310	1.1728	0.2355	0.149*	
H7B	0.4502	1.1812	0.2574	0.149*	
H7C	0.5302	1.0830	0.3061	0.149*	
C9	0.6186 (3)	1.3730 (5)	0.3377 (3)	0.1022 (15)	
H9	0.638 (2)	1.383 (4)	0.298 (2)	0.123*	
C10	0.6447 (2)	1.4513 (4)	0.4071 (3)	0.0998 (15)	
C11	0.7108 (2)	1.5670 (4)	0.4364 (3)	0.1496 (18)	
H11A	0.7145	1.6011	0.4871	0.224*	
H11B	0.6915	1.6386	0.3966	0.224*	
H11C	0.7685	1.5349	0.4443	0.224*	

### Atomic displacement parameters (Å^2^)

**Table:**

	*U*^11^	*U*^22^	*U*^33^	*U*^12^	*U*^13^	*U*^23^
P1	0.0495 (5)	0.0512 (5)	0.0563 (5)	0.0002 (4)	0.0221 (4)	−0.0042 (4)
O3	0.0573 (12)	0.0545 (13)	0.0719 (13)	−0.0055 (11)	0.0194 (10)	−0.0128 (10)
C12	0.0508 (19)	0.051 (2)	0.060 (2)	0.0008 (15)	0.0264 (17)	−0.0001 (16)
C13	0.070 (2)	0.061 (2)	0.075 (2)	−0.0039 (19)	0.023 (2)	0.0053 (19)
C14	0.073 (3)	0.078 (3)	0.091 (3)	−0.002 (2)	0.013 (2)	0.023 (2)
C15	0.067 (2)	0.067 (3)	0.115 (3)	0.013 (2)	0.039 (2)	0.026 (2)
C16	0.085 (3)	0.059 (3)	0.106 (3)	0.012 (2)	0.056 (2)	0.001 (2)
C17	0.071 (2)	0.061 (2)	0.075 (2)	0.0125 (19)	0.0339 (19)	0.002 (2)
C18	0.0521 (17)	0.0464 (19)	0.0552 (17)	−0.0014 (17)	0.0259 (14)	−0.0045 (16)
C19	0.066 (2)	0.060 (2)	0.088 (2)	−0.0017 (18)	0.0424 (19)	−0.0111 (18)
C20	0.071 (2)	0.066 (3)	0.112 (3)	−0.010 (2)	0.049 (2)	−0.009 (2)
C21	0.063 (2)	0.080 (3)	0.106 (3)	−0.001 (2)	0.048 (2)	−0.004 (2)
C22	0.079 (3)	0.071 (3)	0.134 (3)	0.010 (2)	0.064 (2)	−0.011 (2)
C23	0.070 (2)	0.056 (2)	0.106 (3)	−0.0042 (19)	0.050 (2)	−0.007 (2)
C24	0.0568 (19)	0.0489 (19)	0.0603 (19)	−0.0006 (17)	0.0288 (17)	−0.0004 (16)
C25	0.069 (2)	0.071 (2)	0.072 (2)	0.0076 (19)	0.0368 (18)	0.0017 (19)
C26	0.099 (3)	0.073 (2)	0.083 (3)	0.010 (2)	0.059 (2)	0.003 (2)
C27	0.114 (3)	0.082 (3)	0.063 (2)	−0.008 (2)	0.044 (3)	0.0024 (19)
C28	0.081 (3)	0.118 (3)	0.063 (3)	−0.008 (2)	0.024 (2)	0.019 (2)
C29	0.063 (2)	0.088 (3)	0.067 (2)	0.0002 (19)	0.0297 (19)	0.0139 (18)
O1	0.0828 (15)	0.0717 (15)	0.0631 (14)	−0.0256 (12)	0.0267 (12)	−0.0080 (12)
C1	0.053 (2)	0.054 (2)	0.064 (2)	−0.0020 (17)	0.0309 (18)	0.0027 (19)
O2	0.1069 (18)	0.0782 (18)	0.0849 (16)	−0.0368 (15)	0.0353 (13)	0.0044 (14)
C2	0.0451 (18)	0.0485 (19)	0.055 (2)	−0.0011 (15)	0.0213 (16)	0.0003 (15)
N1	0.0491 (15)	0.0534 (17)	0.0658 (17)	−0.0057 (13)	0.0262 (14)	0.0016 (13)
C3	0.055 (2)	0.072 (2)	0.062 (2)	−0.0038 (18)	0.0289 (17)	0.0020 (18)
Cl1	0.0865 (6)	0.1195 (8)	0.0837 (6)	−0.0107 (6)	0.0526 (5)	0.0067 (5)
C4	0.076 (2)	0.103 (3)	0.069 (2)	−0.014 (2)	0.030 (2)	−0.018 (2)
C6	0.0494 (19)	0.054 (2)	0.076 (2)	−0.0038 (17)	0.0248 (18)	0.005 (2)
N2	0.0549 (17)	0.061 (2)	0.098 (2)	−0.0123 (14)	0.0318 (17)	0.0078 (17)
C5	0.069 (2)	0.085 (3)	0.085 (3)	−0.023 (2)	0.027 (2)	−0.023 (2)
N3	0.067 (2)	0.059 (2)	0.149 (3)	−0.0130 (16)	0.044 (2)	−0.0033 (19)
C8	0.059 (2)	0.084 (3)	0.098 (3)	−0.001 (2)	0.035 (2)	0.033 (2)
C7	0.089 (3)	0.140 (4)	0.075 (2)	−0.018 (3)	0.041 (2)	0.011 (2)
C9	0.070 (3)	0.104 (4)	0.136 (5)	0.001 (3)	0.049 (3)	0.050 (3)
C10	0.062 (3)	0.065 (3)	0.171 (5)	−0.003 (2)	0.050 (3)	0.030 (3)
C11	0.094 (3)	0.076 (3)	0.276 (6)	−0.028 (3)	0.079 (3)	0.018 (3)

### Geometric parameters (Å, °)

**Table:**

P1—O3	1.4887 (17)	C27—H27	0.9300
P1—C24	1.779 (3)	C28—C29	1.379 (4)
P1—C12	1.786 (3)	C28—H28	0.9300
P1—C18	1.794 (3)	C29—H29	0.9300
C12—C17	1.376 (4)	O1—C1	1.301 (3)
C12—C13	1.378 (3)	O1—H1	0.7941
C13—C14	1.384 (4)	C1—O2	1.193 (3)
C13—H13	0.9300	C1—C2	1.492 (4)
C14—C15	1.356 (4)	C2—N1	1.339 (3)
C14—H14	0.9300	C2—C3	1.366 (3)
C15—C16	1.364 (4)	N1—C6	1.320 (3)
C15—H15	0.9300	C3—C4	1.385 (4)
C16—C17	1.378 (4)	C3—Cl1	1.725 (3)
C16—H16	0.9300	C4—C5	1.344 (4)
C17—H17	0.9300	C4—H4A	0.9300
C18—C23	1.364 (3)	C6—C5	1.385 (4)
C18—C19	1.371 (3)	C6—N2	1.400 (3)
C19—C20	1.385 (3)	N2—N3	1.377 (3)
C19—H19	0.9300	N2—C8	1.379 (4)
C20—C21	1.355 (4)	C5—H5A	0.9300
C20—H20	0.9300	N3—C10	1.330 (4)
C21—C22	1.351 (4)	C8—C9	1.348 (5)
C21—H21	0.9300	C8—C7	1.483 (4)
C22—C23	1.391 (4)	C7—H7A	0.9600
C22—H22	0.9300	C7—H7B	0.9600
C23—H23	0.9300	C7—H7C	0.9600
C24—C29	1.378 (3)	C9—C10	1.379 (5)
C24—C25	1.386 (3)	C9—H9	0.93 (3)
C25—C26	1.380 (3)	C10—C11	1.507 (5)
C25—H25	0.9300	C11—H11A	0.9600
C26—C27	1.358 (4)	C11—H11B	0.9600
C26—H26	0.9300	C11—H11C	0.9600
C27—C28	1.356 (4)		
			
O3—P1—C24	112.01 (12)	C26—C27—H27	119.7
O3—P1—C12	112.87 (12)	C27—C28—C29	120.2 (3)
C24—P1—C12	106.73 (14)	C27—C28—H28	119.9
O3—P1—C18	110.91 (12)	C29—C28—H28	119.9
C24—P1—C18	106.58 (13)	C24—C29—C28	120.6 (3)
C12—P1—C18	107.40 (13)	C24—C29—H29	119.7
C17—C12—C13	118.3 (3)	C28—C29—H29	119.7
C17—C12—P1	123.1 (2)	C1—O1—H1	113.8
C13—C12—P1	118.5 (2)	O2—C1—O1	123.5 (3)
C12—C13—C14	120.3 (3)	O2—C1—C2	123.7 (3)
C12—C13—H13	119.8	O1—C1—C2	112.8 (3)
C14—C13—H13	119.8	N1—C2—C3	121.7 (3)
C15—C14—C13	120.6 (3)	N1—C2—C1	115.2 (3)
C15—C14—H14	119.7	C3—C2—C1	123.0 (3)
C13—C14—H14	119.7	C6—N1—C2	118.8 (3)
C14—C15—C16	119.7 (4)	C2—C3—C4	118.7 (3)
C14—C15—H15	120.1	C2—C3—Cl1	123.4 (3)
C16—C15—H15	120.1	C4—C3—Cl1	117.8 (3)
C15—C16—C17	120.1 (3)	C5—C4—C3	119.7 (3)
C15—C16—H16	119.9	C5—C4—H4A	120.1
C17—C16—H16	119.9	C3—C4—H4A	120.1
C12—C17—C16	120.9 (3)	N1—C6—C5	122.5 (3)
C12—C17—H17	119.5	N1—C6—N2	116.6 (3)
C16—C17—H17	119.5	C5—C6—N2	120.9 (3)
C23—C18—C19	118.8 (3)	N3—N2—C8	112.1 (3)
C23—C18—P1	117.9 (2)	N3—N2—C6	117.4 (3)
C19—C18—P1	123.3 (2)	C8—N2—C6	130.5 (3)
C18—C19—C20	120.5 (3)	C4—C5—C6	118.6 (3)
C18—C19—H19	119.7	C4—C5—H5A	120.7
C20—C19—H19	119.7	C6—C5—H5A	120.7
C21—C20—C19	119.6 (3)	C10—N3—N2	103.4 (3)
C21—C20—H20	120.2	C9—C8—N2	104.8 (4)
C19—C20—H20	120.2	C9—C8—C7	129.3 (5)
C22—C21—C20	120.9 (3)	N2—C8—C7	125.8 (3)
C22—C21—H21	119.5	C8—C7—H7A	109.5
C20—C21—H21	119.5	C8—C7—H7B	109.5
C21—C22—C23	119.4 (3)	H7A—C7—H7B	109.5
C21—C22—H22	120.3	C8—C7—H7C	109.5
C23—C22—H22	120.3	H7A—C7—H7C	109.5
C18—C23—C22	120.7 (3)	H7B—C7—H7C	109.5
C18—C23—H23	119.7	C8—C9—C10	107.9 (4)
C22—C23—H23	119.7	C8—C9—H9	126 (2)
C29—C24—C25	118.0 (3)	C10—C9—H9	126 (2)
C29—C24—P1	122.9 (2)	N3—C10—C9	111.7 (4)
C25—C24—P1	118.9 (2)	N3—C10—C11	119.0 (5)
C26—C25—C24	121.0 (3)	C9—C10—C11	129.3 (5)
C26—C25—H25	119.5	C10—C11—H11A	109.5
C24—C25—H25	119.5	C10—C11—H11B	109.5
C27—C26—C25	119.6 (3)	H11A—C11—H11B	109.5
C27—C26—H26	120.2	C10—C11—H11C	109.5
C25—C26—H26	120.2	H11A—C11—H11C	109.5
C28—C27—C26	120.7 (3)	H11B—C11—H11C	109.5
C28—C27—H27	119.7		
			
O3—P1—C12—C17	−152.2 (2)	C26—C27—C28—C29	0.2 (5)
C24—P1—C12—C17	−28.7 (3)	C25—C24—C29—C28	0.2 (5)
C18—P1—C12—C17	85.2 (3)	P1—C24—C29—C28	175.2 (2)
O3—P1—C12—C13	29.6 (3)	C27—C28—C29—C24	−0.3 (5)
C24—P1—C12—C13	153.1 (2)	O2—C1—C2—N1	147.7 (3)
C18—P1—C12—C13	−92.9 (2)	O1—C1—C2—N1	−32.9 (3)
C17—C12—C13—C14	0.2 (4)	O2—C1—C2—C3	−33.1 (4)
P1—C12—C13—C14	178.5 (2)	O1—C1—C2—C3	146.3 (3)
C12—C13—C14—C15	0.8 (5)	C3—C2—N1—C6	1.8 (4)
C13—C14—C15—C16	−1.8 (5)	C1—C2—N1—C6	−179.0 (2)
C14—C15—C16—C17	1.7 (5)	N1—C2—C3—C4	−0.3 (4)
C13—C12—C17—C16	−0.3 (4)	C1—C2—C3—C4	−179.4 (3)
P1—C12—C17—C16	−178.5 (2)	N1—C2—C3—Cl1	177.3 (2)
C15—C16—C17—C12	−0.6 (5)	C1—C2—C3—Cl1	−1.9 (4)
O3—P1—C18—C23	19.5 (3)	C2—C3—C4—C5	−0.7 (5)
C24—P1—C18—C23	−102.6 (2)	Cl1—C3—C4—C5	−178.4 (3)
C12—P1—C18—C23	143.3 (2)	C2—N1—C6—C5	−2.4 (4)
O3—P1—C18—C19	−160.2 (2)	C2—N1—C6—N2	179.0 (2)
C24—P1—C18—C19	77.6 (3)	N1—C6—N2—N3	169.7 (2)
C12—P1—C18—C19	−36.5 (3)	C5—C6—N2—N3	−8.9 (4)
C23—C18—C19—C20	0.4 (4)	N1—C6—N2—C8	−12.4 (4)
P1—C18—C19—C20	−179.8 (2)	C5—C6—N2—C8	168.9 (3)
C18—C19—C20—C21	0.9 (5)	C3—C4—C5—C6	0.2 (5)
C19—C20—C21—C22	−1.0 (5)	N1—C6—C5—C4	1.4 (5)
C20—C21—C22—C23	−0.1 (5)	N2—C6—C5—C4	180.0 (3)
C19—C18—C23—C22	−1.6 (5)	C8—N2—N3—C10	0.8 (3)
P1—C18—C23—C22	178.7 (2)	C6—N2—N3—C10	179.1 (3)
C21—C22—C23—C18	1.4 (5)	N3—N2—C8—C9	−1.0 (4)
O3—P1—C24—C29	−104.1 (3)	C6—N2—C8—C9	−178.9 (3)
C12—P1—C24—C29	131.9 (3)	N3—N2—C8—C7	177.7 (3)
C18—P1—C24—C29	17.3 (3)	C6—N2—C8—C7	−0.2 (5)
O3—P1—C24—C25	70.8 (3)	N2—C8—C9—C10	0.8 (4)
C12—P1—C24—C25	−53.2 (3)	C7—C8—C9—C10	−177.9 (3)
C18—P1—C24—C25	−167.7 (2)	N2—N3—C10—C9	−0.3 (4)
C29—C24—C25—C26	0.0 (4)	N2—N3—C10—C11	−179.4 (3)
P1—C24—C25—C26	−175.2 (2)	C8—C9—C10—N3	−0.3 (5)
C24—C25—C26—C27	−0.1 (5)	C8—C9—C10—C11	178.7 (3)
C25—C26—C27—C28	0.0 (5)		

### Hydrogen-bond geometry (Å, °)

**Table:**

*D*—H···*A*	*D*—H	H···*A*	*D*···*A*	*D*—H···*A*
O1—H1···O3	0.79	1.76	2.537 (2)	165
